# Factors associated with professional identity formation within psychiatry residency training: A longitudinal study

**DOI:** 10.1007/s40037-021-00673-w

**Published:** 2021-07-07

**Authors:** Qian Hui Chew, Yvonne Steinert, Kang Sim

**Affiliations:** 1 Research Division, Institute of Mental Health, Singapore, Singapore; 2 Institute of Health Sciences Education, Faculty of Family Medicine & Health Science, McGill University, Montreal, Quebec Canada; 3 National Psychiatry Residency Program and West Region, Institute of Mental Health, Singapore, Singapore

**Keywords:** Professional identity formation, Residency, Psychiatry

## Abstract

**Introduction:**

Conceptual frameworks for professional identity (PI) formation highlight the importance of developmental stages and socialization as the learner progresses from legitimate peripheral to full participation. Based on extant literature and clinical impressions, the authors aimed to explore factors associated with PI formation in psychiatry residents over time, and hypothesized that time in training, seniority status, and duration of exposure to psychiatry prior to residency would be associated with PI formation.

**Methods:**

Eighty out of 96 psychiatry residents (response rate, 83.3%) from the National Psychiatry Residency Program in Singapore participated and rated their PI development using the Professional Self Identity Questionnaire (PSIQ) across four timepoints from January 2016–December 2019. The residents were classified as junior (first 3 years) or senior residents (years 4–5). Linear mixed model analyses were conducted, with time in training, seniority status (junior versus senior residents), duration of psychiatry postings prior to residency, and their interaction as associated factors with PI over time.

**Results:**

Time in training, seniority, and duration of psychiatry postings before residency (all *p* < 0.01) were significantly associated with higher PSIQ scores at baseline. Over time, although all residents had increases in PSIQ scores, this rate of change did not differ significantly between junior and senior residents.

**Discussion:**

Exposure to psychiatry postings before residency, time in learning, and seniority are factors which influence PI development in residents. This has implications for psychiatry residency selection and training, adequate clinical exposure during training rotations, and continual support for new and senior residents to foster PI formation over time.

**Supplementary Information:**

The online version of this article (10.1007/s40037-021-00673-w) contains supplementary material, which is available to authorized users.

## Introduction

Professional identity (PI) formation, defined as the development of professional values, actions, and aspirations, has been deemed as the backbone of medical education [1]. As residents prepare for the transition to full-fledged physicians throughout the course of residency, one determinant of their competence and success is the formation of a PI [2]. When a physician’s personal and professional identities are incongruent, problems may arise for the clinician, the patient, and the profession. First, the clinician may experience identity dissonance when their personal and professional identities are perceived to be poorly aligned with each other [2–5]. The inability to reconcile these identities could result in emotional difficulties, and uncertainty about values, competence, and even self-worth [2]. Additionally, Abedini and colleagues [6] have highlighted the issue of uncertainty in one’s PI and its contribution to burnout as well as one’s ability to recover from it. The patients’ confidence is also likely to waver when the clinician is unable to project a sense of confidence, competency, and professionalism as a result of problems with PI formation [7]. This may hinder the building of therapeutic alliance, which could affect treatment outcomes and patients’ adherence to treatment. The profession and/or subspecialty’s reputation could be negatively affected by poor PI formation and ethical breaches. One measure of a well-formed PI is the internalization of ethical rules relevant to the profession [8]. An incomplete or disrupted formation of PI could result in ethical standards being viewed as rules external to the self and subsequent difficulty in self-regulation related to one’s own practice [9]. Thus, we need to examine the formation of PI as well as factors that influence its formation to help residency programs support residents in becoming well-developed clinicians. These findings would also have utility across residencies which seek to understand and support the development of PI within their learners and better delineate the factors which influence this process.

There is generally a paucity of studies examining the formation of PI longitudinally in the context of psychiatry. Current longitudinal studies regarding PI formation in residency have mostly emerged from family medicine and surgical disciplines. In addition, disagreements remain concerning specific factors that could influence its growth trajectory [10–13]. Stages in PI development can be likened to stages in human development (e.g., Erikson’s stages of psychosocial development), where residents would attempt to establish autonomy through independent clinical decision making guided by seniors [11]. A failure to progress could mean that residents fail to develop a confident decision-making style and remain stuck in the stage of autonomy versus shame and doubt [11]. Studies also suggest that the initial desire for a role model would later evolve into a desire to be a good role model for others as residents gained more clinical experience and seniority [10]. A well-formed PI was associated with a desire to contribute to the advancement of their specialty [10], enhanced competence, and the ability to lead others [11, 12].

Nonetheless, the literature also suggested evidence of potential problems that accompanied the development of PI, such as an erosion of personal relationships [14] and emotional detachment [13]. Despite several studies showing changes in PI over time, there have also been mixed findings, with Monrouxe and colleagues [15] reporting that PI was actually stable over time as final-year medical students transitioned into clinical practice. A longitudinal qualitative study conducted with internal medicine residents found that professional development was the most frequently reported concern for residents from PGY1 to PGY3, with the exception of the final trimester of PGY3 when residents reported developing better coping skills [16]. In addition, it has also been noted that those with greater profession-related pre-program experience as well as senior students have a better developed PI [17]. This suggests that factors such as prior clinical exposure to a clinical discipline and time in training may affect PI formation amongst learners in training.

The discipline of psychiatry is unique compared to other medical and surgical disciplines in its strong focus on the holistic evaluation and management of the person using the biopsychosocial model [18], taking a lifetime perspective in view of chronicity of psychiatric conditions [19], and emphasis on interprofessional collaboration [20], all of which are integral to PI formation and all of which take time to inculcate and develop within psychiatry residency training. In addition, psychiatry suffers from the unique problem of stigmatization at various levels. There is a negative perception of psychiatrists on the part of other medical professionals who view it as a non-medical discipline [21]. Psychiatrists are also often faced with stigmatization from caregivers or the public towards their patients, and even patients who experience self-stigmatization [21]. This suggests that a well-formed PI is crucial in encouraging psychiatry residents to be effective advocates not only for their medical specialty, but also for their patients and caregivers—and in this way combating the problem of stigma that surrounds the topic of mental health.

In view of the sparse data on PI formation specific to psychiatry residents in training and conflicting findings, we sought to examine the development of PI over time and factors influencing it amongst our residents. Based on available literature and clinical impressions, we hypothesize that seniority status and duration of psychiatry postings prior to residency influence PI formation within psychiatry residents.

## Methods

### Study sample

All psychiatry residents from the National Psychiatry Residency Program in Singapore were invited to participate in this longitudinal study from January 2016–December 2017, and we followed up recruited participants until December 2019. The National Psychiatry Residency Program is a five-year training program which is accredited by the Accreditation Council of Graduate Medical Education–International (ACGME–I) and is the only psychiatry residency program in Singapore. It has been centrally co-ordinated by the National Healthcare Group Residency Office since 2010. There is capacity to take up to 80 residents. Residents from local and international medical schools are accepted into this program. The residency program has a rigorous curriculum and rotations across different training sites covering areas such as General Psychiatry and various subspecialties such as Child and Adolescent Psychiatry, Geriatric Psychiatry, Consultation Liaison Psychiatry, Forensic Psychiatry, Emergency Psychiatry as well as electives which residents select in their final year of training. Teaching faculty include clinicians from the diverse training sites.

### Procedures

This study was approved by the Institutional Review Board of the National Healthcare Group, Singapore (NHG DSRB Ref: 2015/01139). All participants provided signed, written informed consent prior to administration of questionnaires. We administered questionnaires to residents at intervals of approximately 6 months over 2 years. Residents in the first 3 years of training were classified as “junior residents,” while residents in years 4 and 5 were classified as “senior residents.” All responses were collected anonymously, including basic demographic data (gender, age, marital status, current year in residency, duration of psychiatry postings before joining residency program). Each resident was assigned a unique identification number at the first timepoint of data collection, and only research staff who were not involved in the residency program had access to the database containing these identification numbers.

### Measure

We assessed the development of participants’ PI using the Professional Self Identity Questionnaire (PSIQ) [17]. It examines the level of PI healthcare professionals experience when undertaking activities such as communicating with patients, working with other health professionals, considering ethical issues, and teaching others [17]. It can be used both cross-sectionally and longitudinally to examine the development profile of those in health and social care professions [17]. The scale consists of nine questions on a six-point Likert scale. A total PSIQ score was obtained by summing up all items, with a higher score indicating better development of PI (minimum score 9, maximum score 54).

The PSIQ has been administered to a sample consisting of healthcare personnel in training and has demonstrated sufficient validity in terms of internal structure and relationships with external variables [17].

### Statistical analyses

We conducted all analyses using IBM SPSS 23 (IBM Corp, Armonk, NY) and R Version 3.5.2 (2018). We used Linear mixed models in R to examine changes in PSIQ scores over time, while taking into account other variables of interest, namely seniority status (junior versus senior residents) and duration of psychiatry postings before joining residency. The estimates were chosen to optimize the full maximum likelihood criterion in order to enable comparison of models [22]. The Satterthwaite approximations in the “lmerTest” statistical package [23] were used to compute the significance of the model parameters. Missing data were taken to be missing completely at random (MCAR). When maximum likelihood is used, it has been found that omission of missing data will still provide unbiased estimates under the assumption that data were MCAR [22].

First, we ran an unconditional means model to assess the amount of outcome variation that exists at both the within-subject and between-subject level. This allows us to examine if there is sufficient variation at each level to warrant the addition of factors to attempt to explain this variation [24].

Upon determining that there was sufficient variation that could be explained by other factors, we ran an unconditional growth model to relate with PSIQ score changes over time. Within-subject and between-subject differences were accounted for as random effects. We followed this by testing two more models which included seniority status and duration of psychiatry postings prior to residency as a factor associated with both initial PSIQ scores and its change over time. All three goodness-of-fit statistics [namely, deviance, Akaike information criterion (AIC), and Bayesian information criterion (BIC)] were examined to determine if the new model tested offered a better fit as compared to the previous model. Based on the results, we then narrowed down the factors chosen to obtain a final, more parsimonious model.

## Results

### Sample characteristics (see Table S1)

Overall, 80 out of 96 residents (response rate of 83.3%) participated in this longitudinal study at baseline. The subsequent timepoints consisted of data collected from 71, 50, and 33 residents, respectively. See Table S1 in the Electronic Supplementary Material (ESM) for participant details and variables of interest (PSIQ score, seniority status) at each timepoint. Of the residents, 61.3% were junior residents at baseline.

### Linear mixed models (see Table 1)

#### Unconditional means model (No predictors)

**Table 1:** Results of fitting a taxonomy of multilevel models for change in Professional Self Identity Questionnaire scores over four timepoints (January 2016–December 2019) for psychiatry residents

	Parameter	Unconditional means model	Unconditional growth model	Intermediate model 1	Intermediate model 2	Final model
		Parameter estimate (*SE*)	Parameter estimate (*SE*)	Parameter estimate (*SE*)	Parameter estimate (*SE*)	Parameter estimate (*SE*)
Fixed Effects						
	Intercept	39.8443*** (0.7597)	38.4320*** (0.8184)	28.8416*** (1.7508)	27.1920*** (1.7720)	28.0927*** (1.5140)
	Time		1.4323*** (0.2407)	1.7700* (0.7826)	2.0148* (0.8775)	1.1945*** (0.2540)
	Junior/Senior			6.8699*** (1.1653)	7.0496*** (1.1309)	6.5025*** (0.9146)
	Time*Junior/Senior			−0.4099 (0.5323)	−0.4296 (0.5354)	
	Duration of psychiatry posting				1.2341** (0.4628)	1.1236* (0.4319)
	Time*Duration of psychiatry posting				−0.1890 (0.2581)	
Variance Components						
	Within-person	13.91 (3.730)	10.3350 (3.2148)	11.1938 (3.3457)	11.2038 (3.3472)	11.2556 (3.3549)
	In initial status	40.56 (6.368)	45.6030 (6.7530)	25.5873 (5.0584)	22.6457 (4.7587)	23.2198 (4.8187)
	In rate of change		0.5907 (0.7686)	0.9573 (0.9784)	0.8888 (0.9428)	0.8068 (0.8982)
	Covariance		−0.44	−0.58	−0.53	−0.55
Goodness-of-fit						
	Deviance	1455.2	1419.0	1388.1	1381.2	1382.2
	AIC	1461.2	1431.0	1404.1	1401.2	1398.2
	BIC	1471.6	1451.7	1431.7	1435.7	1425.8

*Note*: Full maximum likelihood (ML) estimation

*AIC* Akaike Information Criterion, *BIC* Bayesian Information Criterion

*p < 0.05, **p < 0.01, ***p < 0.001

We first tested an unconditional means model which describes the within-person and between-person variances in the absence of any predictors. There were 234 observations obtained from 80 residents used in the model. Based on the between- and within-person variances, we calculated the intraclass correlation coefficient, which indicated that 74.5% of the variance was the result of between-person differences.

#### Unconditional growth model (Predictor: time)

Next, we fitted an unconditional growth model, which introduces the predictor of time into the model, to explore changes in PI over time. Based on the results, we were able to conclude that the average change trajectory for PSIQ scores had an intercept of 38.4 and a slope of +1.43, which differ significantly from zero (both *p* < 0.001). The plot for this model is given in Fig. S1 in the ESM. The three goodness-of-fit indices (deviance, AIC, BIC) indicated that the addition of time as a factor resulted in a better fitting model as compared to the unconditional means model. Additionally, 18.3% of the within-person variation in PSIQ scores was systematically associated with the factor time.

#### Intermediate model 1 (Predictors: time, seniority status, time*seniority status)

We then ran models with the addition of our factors of interest. First, seniority status, time, and its interaction term were added into the model. This would allow us to explore differences in PI between junior and senior residents at baseline, its changes over time, as well as differences in the rate of change between both groups. Based on the results, the estimated initial PSIQ score for the average junior resident was 28.8 (*p* < 0.001). The average difference between initial PSIQ scores for senior and junior residents was 6.87 (*p* < 0.001). The estimated rate of change in PSIQ scores for an average junior resident was 1.77 (*p* < 0.05). Nonetheless, the estimated difference in rate of change of PSIQ scores between junior and senior residents was not significant (−0.41, ns). Given the better goodness-of-fit statistics (deviance, AIC, BIC), seniority status is a significant predictor of PSIQ scores and produces a better fitting model.

#### Intermediate model 2 (Predictors: time, seniority status, time*seniority status, duration of psychiatry postings prior to residency, time*duration of psychiatry postings prior to residency)

Second, duration of psychiatry postings prior to residency and its interaction term with time were added into the previous model. This would allow us to examine whether duration of psychiatry postings prior to residency influenced PI at baseline, as well as whether those with a longer duration of psychiatry postings prior to residency had differences in rate of PI development over time as compared to those with a shorter duration of psychiatry postings prior to residency. Based on the results, the estimated initial PSIQ score for the average junior resident with duration of psychiatry postings prior to residency of 0 years was 27.2 (*p* < 0.001). The estimated rate of change in PSIQ scores for an average junior resident with duration of psychiatry postings prior to residency of 0 years was 2.01 (*p* < 0.05). Controlling for the effects of duration of psychiatry postings prior to residency, the estimated differential in initial PSIQ scores between junior and senior residents was 7.05 (*p* < 0.001). Just as in the previous model, the estimated difference in rate of change of PSIQ scores for junior and senior residents did not reach statistical significance (−0.43, ns). Given the significant fixed effects of duration of psychiatry postings prior to residency (1.23, *p* < 0.01) and the better goodness-of-fit statistics (deviance and AIC), duration of psychiatry postings prior to residency is a significant factor associated with PSIQ scores in addition to seniority status and produces a better fitting model.

#### Final model (Predictors: time, seniority status, duration of psychiatry postings prior to residency)

Our final parsimonious model included only the significant factors obtained from previous models tested (time, seniority status and duration of psychiatry postings prior to residency). Controlling for the effects of duration of psychiatry postings prior to residency, the estimated difference in initial scores between junior and senior residents was 6.50 (*p* < 0.001). Controlling for the effects of seniority status, for each 1‑year difference in duration of psychiatry postings prior to residency, the average initial PSIQ score was 1.12 points higher (*p* < 0.05).

## Discussion

Overall, we observed several findings. First, there was a significant and positive change in PI formation over time. Second, seniority and duration of psychiatry postings before residency were significant independent factors associated with better PI formation at baseline. Third, although both junior and senior residents had enhanced PI over time, these two groups did not differ significantly in their rates of this positive change.

We found that the PSIQ scores improved over time for all residents, which is in agreement with various stage-based models of PI development that describe better PI formation over time [3, 25]. Stages 2–4 are most relevant to a medical professional and begin with individuals taking on the role of a clinician by following the rules and performing behaviour that is expected of them and involves little self-reflection. Stage 3 represents an individual who begins the process of self-reflection and integration with the profession. Finally, stage 4 is characterized by an individual who is not only able to incorporate their personal core values with that of the profession, but also reconcile any conflicts that may arise. This is likened to the level of “being” beyond “showing how” and “doing,” in which the clinician is able to navigate ambiguities inherent in medical practice and consistently respond as a professional within the clinical profession or specialty [25, 26]. Over time, the immersion in training encourages the appropriate development of professional self-identity [17] and addresses concerns that learners may have about their own PI [16].

Hence, suggested reasons for the positive change in PSIQ scores include increased perceived competence as physicians, as well as having a clearer idea of future plans and goals [16]. Not only do residents progress from “knowing” and “knowing how” to actively “doing” things expected of a physician [25, 26], they also transition into the “interpersonal” stage of professional development and may be more confident as they reflect on their future plans [3, 27]. Additionally, learners have also cited incremental patient encounters over time as a main factor influencing PI development [5]. The time spent in learning over time offers residents more opportunities to learn from patients, reflect on clinical encounters, and navigate the balance between empathy and efficiency in practice.

For seniority status, senior residents had higher initial PSIQ scores than junior residents at baseline, which is consistent with previous findings albeit within student doctors [17]. The difference in PSIQ scores at baseline can be explained by the level of participation in their community of practice at various stages of the residency program [28, 29]. Early on in residency, junior residents are still acquiring the knowledge necessary for clinical practice [25, 26], conducting clinical evaluation skills under supervision, and beginning to be socialized into the profession from a standpoint of legitimate peripheral participation [28, 29]. On the other hand, senior residents are given more role autonomy and responsibilities, which allow them to transition from legitimate peripheral participation to full participation in their community of practice [28, 29]. As their clinical decisions have an increasing influence on patient care, greater self-reflection about their practice would ensue, which in turn would aid the formation of PI amongst the senior residents [3], hence explaining the higher PSIQ scores amongst the senior versus the junior residents. Apart from greater involvement in clinical practice, senior residents are also encouraged to take on mentoring roles and help junior residents better integrate into the program. This transition is likely to prompt them to seek out role models or attempt to emulate those they have encountered previously, further anchoring their PI at the “interpersonal” stage of development [3].

The improvement in PSIQ scores did not differ significantly between junior and senior residents over time, suggesting comparable rates of growth in PI longitudinally once enrolled in the residency training program. This could indicate that there are sufficient and appropriate opportunities in each residency group to facilitate the development of PI longitudinally. For junior residents, there is a greater focus on developing competencies in medical knowledge and patient care, which is then evaluated through formative assessments by faculty, ensuring that they attain the competencies alongside socialization into the discipline [3, 25]. As for senior residents, the focus in training shifts towards developing the ability to make independent clinical decisions and to self-regulate one’s own practice [9]. This process prompts them to “think, act, and feel like a physician” and progress to the final stages of PI development in a continual developmental process [25, 30].

Of note, we observed that the duration of psychiatry postings before the start of residency was associated with higher initial PSIQ scores, which suggests that the amount of relevant clinical experience accumulated before residency influences positively the extent of PI development. Relevant clinical experience could facilitate greater participation in one’s community of practice [28, 29], as reflected in a substantial period of being immersed within relevant rotations prior to residency, or seniority. This is supported by findings in previous studies which noted that access to authentic clinical experiences [31] and more profession-related pre-program experience [17] were important contributors to the development of PI.

Based on our findings, we suggest several practical applications for psychiatry and other residency programs to consider. First, selection panels of prospective candidates into psychiatry residency training may want to consider the duration of prior appropriate exposure during the selection process, as this positively influences the development of PI. This could include both formal experience (e.g., time spent in psychiatry clinics/wards) and informal experience (e.g., caregiver of someone with mental health conditions). A holistic assessment of the candidate’s prior exposure and personal experience related to the discipline can be assessed based on portfolio reflections and in-depth interviews. Second, the residency program can be structured to ensure adequate opportunities for patient encounters early in the program. This eases the transition from legitimate peripheral participation to full participation in their community of practice [28, 29] and allows the learners to better appreciate and fulfil the values of the profession in their practice [30]. Third, junior residents are more likely to have concerns and feelings of uncertainty as they attempt to integrate into residency. It is therefore important for both faculty and senior residents to allay these concerns by providing them with better social support [13]. This could come in the form of a buddy system which pairs junior residents with senior residents, with provisions for regular meetings in formal and informal settings to lend an ear [16]. As senior residents share their personal and professional growth and journey within residency, this could also help reassure junior residents that PI formation is a process that takes time to nurture. Additionally, such a mentoring system provides access to role models early in practice [3], and an external yardstick against which one’s own progress in PI formation can be referenced. Furthermore, residents over time can be empowered to participate in guided critical reflection which can facilitate both their personal growth as well as deeper insights into their professional interactions with patients under their care [30, 31]. These sessions, including those between junior and senior residents and between senior residents and faculty or mentors, can help to provide a safe learning environment and help residents navigate conflicts between their personal and professional values whenever they arise. In addition, it may also be posited that the implications of prior clinical experience are important not only for earlier immersion in one’s community of practice for psychiatry residents, but also a key element for the cultivation of empathy and lowering of stigma-related barriers unique to the field of psychiatry.

### Limitations

There are several limitations in this study. First, although the invitation to this study was extended to all psychiatry residents in Singapore, the size of the cohort is still modest and future studies may seek to replicate our findings with a larger cohort. Second, the follow-up length is relatively short and could be extended to understand the trajectory of PI development over a longer time course. Third, other factors influencing PI formation could be further studied including the nature of the learning environment, availability of role models, and the relationship between personal and professional growth with curriculum content and format (such as spaced learning of different topics with faculty support) during residency training.

## Conclusions

Consistent with extant developmental models of PI formation, appropriate exposure to psychiatry postings before entering residency, time in learning, and seniority are factors which can influence PI formation amongst our psychiatry residents. This has implications for psychiatry residency selection, adequate clinical exposure during training rotations, emphasis on continual support for our junior residents by seniors and faculty, and empowerment of senior residents in order to foster better PI formation over time.

## Supplementary Information

**Table S1 **Descriptive statistics across four timepoints (January 2016–December 2019) for psychiatry residents. *PSIQ* Professional Self Identity Questionnaire

**Fig S1 **Unconditional growth model of Professional Self Identity Questionnaire (PSIQ) scores over four timepoints (January 2016–December 2019) for residents from the National Psychiatry Residency Program in Singapore

## References

[1] Cooke M, Irby D, O’Brien BC (2010). Educating physicians: a call for reform of medical school and residency.

[2] Costello CY (2005). Professional identity crisis: race, class, gender, and success at professional schools.

[3] Cruess RL, Cruess SR, Boudreau JD, Snell L, Steinert Y (2015). A schematic representation of the professional identity formation and socialization of medical students and residents: a guide for medical educators. Acad Med.

[4] Monrouxe LV (2010). Identity, identification and medical education: why should we care?. Med Educ.

[5] Wong A, Trollope-Kumar K (2014). Reflections: an inquiry into medical students’ professional identity formation. Med Educ.

[6] Abedini NC, Stack SW, Goodman JL, Steinberg KP (2018). “It’s not just time off”: a framework for understanding factors promoting recovery from burnout among internal medicine residents. J Grad Med Educ.

[7] Freedman DP, Holmes MS (2003). The teacher’s body: embodiment, authority, and identity in the academy.

[8] Cruess RL, Cruess SR, Boudreau JD, Snell L, Steinert Y (2014). Reframing medical education to support professional identity formation. Acad Med.

[9] Wald HS (2015). Professional identity (trans)formation in medical education: reflection, relationship, resilience. Acad Med.

[10] Hansen SE, Mathieu SS, Biery N, Dostal J (2019). The emergence of family medicine identity among first-year residents: a qualitative study. Fam Med.

[11] Lipsman N, Khan O, Kulkarni AV (2017). “The actualized neurosurgeon”: a proposed model of surgical resident development. World Neurosurg.

[12] de Montbrun S, Patel P, Mobilio MH, Moulton CA (2018). Am I cut out for this? Transitioning from surgical trainee to attending. J Surg Educ.

[13] Phillips SP, Dalgarno N (2017). Professionalism, professionalization, expertise and compassion: a qualitative study of medical residents. BMC Med Educ.

[14] Law M, Lam M, Wu D, Veinot P, Mylopoulos M (2017). Changes in personal relationships during residency and their effects on resident wellness: a qualitative study. Acad Med.

[15] Monrouxe LV, Bullock A, Tseng HM, Wells SE (2017). Association of professional identity, gender, team understanding, anxiety and workplace learning alignment with burnout in junior doctors: a longitudinal cohort study. BMJ Open.

[16] Satterfield JM, Becerra C (2010). Developmental challenges, stressors and coping strategies in medical residents: a qualitative analysis of support groups. Med Educ.

[17] Crossley J, Vivekananda-Schmidt P (2009). The development and evaluation of a Professional Self Identity Questionnaire to measure evolving professional self-identity in health and social care students. Med Teach.

[18] Engel GL (1980). The clinical application of the biopsychosocial model. Am J Psychiatry.

[19] Rutter M (1986). Meyerian psychobiology, personality development, and the role of life experiences. Am J Psychiatry.

[20] Marcussen M, Nørgaard B, Borgnakke K, Arnfred S (2020). Improved patient-reported outcomes after interprofessional training in mental health: a nonrandomized intervention study. BMC Psychiatry.

[21] Fiorillo A, Malik A, Luciano M, Del Vecchio V, Sampogna G, Del Gaudio L (2013). Challenges for trainees in psychiatry and early career psychiatrists. Int Rev Psychiatry.

[22] Verbeke G, Molenberghs G (2010). Linear mixed models for longitudinal data.

[23] Kuznetsova A, Brockhoff PB, Christensen RHB (2017). lmerTest package: tests in linear mixed effects models. J Stat Softw.

[24] Singer JD, Willett JB (2009). Applied longitudinal data analysis: modeling change and event occurrence.

[25] Cruess RL, Cruess SR, Steinert Y (2016). Amending Miller’s pyramid to include professional identity formation. Acad Med.

[26] Miller GE (1990). The assessment of clinical skills/competence/performance. Acad Med.

[27] Kegan R (1982). The evolving self.

[28] Lave J, Wenger E (1991). Situated learning: legitimate peripheral participation.

[29] Wenger E (1998). Communities of practice: learning, meaning, and identity.

[30] Irby DM, Hamstra SJ (2016). Parting the clouds: three professionalism frameworks in medical education. Acad Med.

[31] Boudreau JD, Macdonald ME, Steinert Y (2014). Affirming professional identities through an apprenticeship: Insights from a four-year longitudinal case study. Acad Med.

