# Supplementary material for: Factors associated with professional identity formation within psychiatry residency training: A longitudinal study
Source: Perspect Med Educ. 2021 Jul 7;10(5):279–85. doi: 10.1007/s40037-021-00673-w (PMC8505594; doi:10.1007/s40037-021-00673-w)
Supplement: Supplementary file 1 — Table S1 Descriptive statistics across four timepoints (January 2016–December 2019) for psychiatry residents. PSIQ Professional Self Identity Questionnaire [file 40037_2021_673_MOESM1_ESM.docx]

**Table S1** Descriptive statistics across four timepoints (January 2016–December 2019) for psychiatry residents. *PSIQ* Professional Self Identity Questionnaire

| Timepoint | Mean (*SD*) age | % of males | % of junior residents | Mean (*SD*) PSIQ scores |
| --- | --- | --- | --- | --- |
| Baseline | 30.0 (2.37) | 55.0 | 61.3 | 38.2 (6.19) |
| Follow-up 1 | 30.1 (2.04) | 54.9 | 56.3 | 40.1 (5.05) |
| Follow-up 2 | 30.6 (1.90) | 58.0 | 56.0 | 40.9 (4.53) |
| Follow-up 3 | 31.0 (1.67) | 51.5 | 60.6 | 42.0 (4.45) |
