# Supplementary material for: Factors associated with professional identity formation within psychiatry residency training: A longitudinal study
Source: Perspect Med Educ. 2021 Jul 7;10(5):279–85. doi: 10.1007/s40037-021-00673-w (PMC8505594; doi:10.1007/s40037-021-00673-w)
Supplement: Supplementary file 2 — Fig S1 Unconditional growth model of Professional Self Identity Questionnaire (PSIQ) scores over four timepoints (January 2016–December 2019) for residents from the National Psychiatry Residency Program in Singapore [file 40037_2021_673_MOESM2_ESM.docx]

**Fig S1** Unconditional growth model of Professional Self Identity Questionnaire (PSIQ) scores over four timepoints (January 2016–December 2019) for residents from the National Psychiatry Residency Program in Singapore
